# The Unexpected Hidden Danger of Lightning Strike: A Report of a Rare Case of Bladder Injury

**DOI:** 10.7759/cureus.106348

**Published:** 2026-04-02

**Authors:** Anshika Singh, Rayala Sri Krishna, Pradeep K Jena, Varsha Madhavnarayan Totadri, Subrat Mohanty, Harish Chandra Tudu, Sudhansu S Mohanty

**Affiliations:** 1 General Surgery, Kalinga Institute of Medical Sciences, Bhubaneswar, IND; 2 Pediatric Surgery, Kalinga Institute of Medical Sciences, Bhubaneswar, IND; 3 Pediatric Surgery, Vardhman Mahavir Medical College and Safdarjung Hospital, New Delhi, IND; 4 Radiodiagnosis, Kalinga Institute of Medical Sciences, Bhubaneswar, IND

**Keywords:** bladder injury, isolated, lightning strike, multisystem, uncommon

## Abstract

Lightning strikes are a rare but potentially devastating cause of injury, particularly in children. Clinical manifestations range from transient neurological disturbances and cardiac arrhythmias to severe burns, auditory damage, and multisystem trauma. Early recognition and prompt resuscitative measures are essential for improving outcomes, as lightning victims often have a high potential for successful resuscitation if treated rapidly. While musculoskeletal, cardiac, and neurological complications are commonly reported, genitourinary injuries, especially bladder trauma, are exceedingly uncommon and, in fact, underreported. We report an unusual and extremely rare clinical case of a lightning strike victim who presented with an isolated bladder injury.

## Introduction

Lightning strike injuries, while less documented, are a causative factor in thousands of deaths annually worldwide [1]. Lightning exposes the body to over 1,000,000 volts and 10,000-200,000 amperes, which is defined as a high-voltage injury [1,2]. As lightning injury involves only a short exposure period, only a small amount of energy is transferred internally, with the remaining majority flowing externally over the victim's body, a phenomenon called the “flashover” effect [3]. Apart from the electric effect, a major contributory factor to lightning injury, especially internal organ injury, is the extreme temperature of lightning or a blast wave, which causes injury through the explosion of air around the lightning channel [3,4].

The nature of injury depends on multiple factors, including the path of current flow through the body, the duration of contact, clothing worn by the affected individual, contact with the ground, and protective coverings such as rubber slippers or shoes [5]. Neurological and cardiovascular systems are most commonly affected, as nerves and blood vessels offer the pathway of least resistance to lightning [1,5,6]. While these have been well documented in the literature, hollow viscus injuries and bladder injuries have minimal to no data available due to their rarity.

Bladder injuries are uncommon due to an unlikely pathway for current and the thick wall of the bladder [3]. If they occur, they are most commonly attributable to falls following a lightning strike and are associated with pelvic fractures, which subsequently cause bladder injury [1-3,6]. Symptoms include urinary retention, dysuria, and hematuria. Bladder injury has not been documented, particularly in the pediatric age group, in the literature.

Given the paucity of data, the significance of this case stems from the isolated bladder injury, which became an unexpected finding. This case report, therefore, highlights the nuances of suspicion, resuscitation, diagnosis, and management of the unlikely bladder injury secondary to a lightning strike and aims to provide guidance on the possible physical mechanisms contributing to the injury and the appropriate management.

## Case presentation

**Figure 1:**
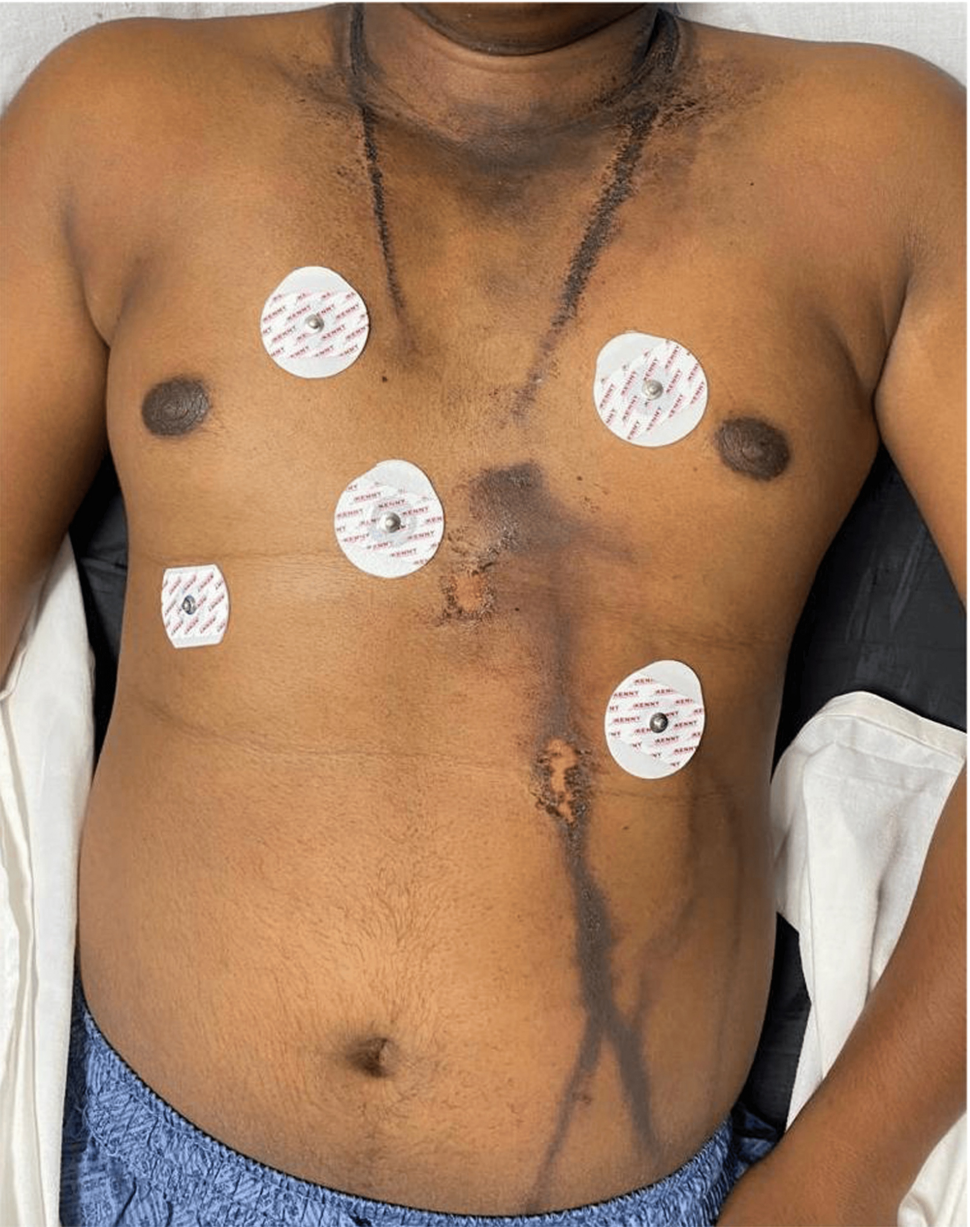
Arborizing pattern of lightning strike over the patient’s torso

A 16-year-old male presented to the pediatric casualty with an alleged history of lightning strike injury during an outdoor event three days prior in a field. The child was initially managed at a peripheral hospital with intravenous fluids and observation. Initial assessment of the casualty revealed a vitally stable patient with a superficial cutaneous burn in a fringed, arborizing pattern, corresponding to the path of current entry and exit, with the entry point near the nape of the neck and the exit point atypically noted near the left iliac crest (Figure 1).

The patient had no evidence of arrhythmia or neurological deficits. On further examination, scleritis and traumatic rupture of the right tympanic membrane were identified. During the initial assessment, his abdomen was soft and non-tender. However, he subsequently developed lower abdominal pain and hematuria on the second day of admission (four days after the lightning strike). The patient was catheterized, and a radiological evaluation was performed. Baseline sonography revealed normal kidneys and ureters but a suspicious defect in the anterior wall of the bladder, with no free fluid in the abdomen.

**Figure 2:**
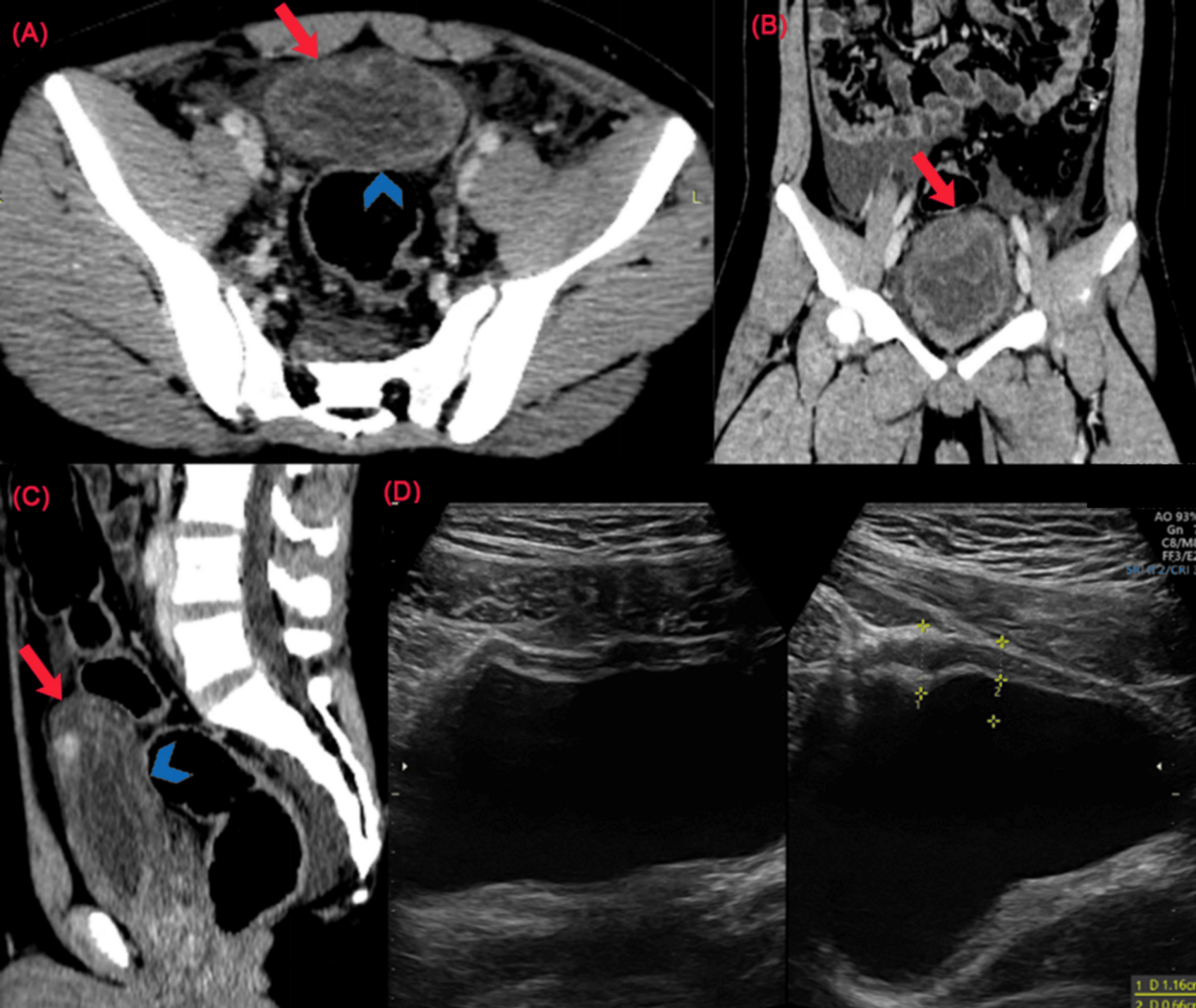
(A-C) Axial, coronal, and sagittal sections of contrast-enhanced CT abdomen and pelvis. (A) Axial section showing diffusely edematous thickening of the urinary bladder wall, predominantly in the superior and posterior aspects (blue arrow). (B) Coronal section showing a focal partial defect (red arrow) in the anterior superior bladder wall. (C) Sagittal section showing diffusely edematous thickening of the superior and posterior bladder wall (blue arrowhead) with a focal partial defect (red arrow) in the anterior superior bladder wall. (D) Follow-up ultrasonography of the abdomen and pelvis showing homogeneous focal thickening of the anterior superior bladder wall without any obvious defect, suggesting resolution in progress.

Contrast-enhanced CT of the abdomen and pelvis, performed on the second day of admission, revealed a 4 mm partial tear of the anterior bladder wall without associated pelvic fractures and no other visceral or solid organ injuries (Figure 2A-2C).

The patient underwent multidisciplinary management in the pediatric intensive care unit, including conservative management of the bladder injury with catheterization. Thorough neurological and cardiac monitoring was performed, along with biochemical and hematological evaluation consistent with the pathology. The patient gradually improved; hematuria resolved, and the catheter was removed one week after insertion (day 9 of admission). He was discharged after a multidisciplinary review 12 days after admission (15 days after the lightning strike). The child remains on regular follow-up over a duration of one year and has shown complete clinical and radiological recovery (Figure 2D).

## Discussion

Bladder injury secondary to a lightning strike is extremely rare and remains undocumented in the literature in children and adolescents. It likely results from a combination of thermal injury and the blast effects of lightning energy [1,2]. When identified, it is commonly associated with pelvic fracture (due to a fall of the victim) or with sudden muscle hypercontractility resulting from lightning energy and voltage [3]. Lightning delivers an extremely high-voltage current over a very brief duration, generating both electrical and mechanical forces. When the bladder is full at the moment of exposure, the contained urine can act as a medium that rapidly expands due to sudden heating. This “steam-driven” expansion may create sufficient internal pressure to rupture the bladder wall [1-3].

Additionally, intense muscle tetany from the electrical current can produce abrupt contraction of the abdominal and pelvic musculature, further increasing intravesical pressure [4,5]. These physical interactions of lightning could possibly explain the mechanism of injury in this particular case. The absence of associated trauma in this adolescent highlights the unique pathophysiology of lightning-related injuries.

It is evident that a deeper understanding of the physics and pathway of current is required to analyze rare injuries such as hollow viscus organ and bladder injuries, which are atypical because they do not form a pathway of least resistance [1,2,6]. In this patient, the arborizing pathway of the lightning took an atypical route with respect to the exit point, which is normally directed toward the feet or another organ in contact with the earth. Here, the exit point was identified near the left iliac fossa, which could indicate the underlying bladder injury.

It is imperative to understand the mechanisms of injury, including direct electrical damage, barotrauma, and reflex detrusor contraction, as this informs clinical suspicion and ensures continued medical examination for delayed signs of injury [1,3,4,6]. Unlike blunt trauma-related bladder rupture, lightning-induced bladder injuries may occur without external evidence of pelvic or abdominal impact, making diagnosis more challenging, as seen in this patient [5,6].

Knowledge of the physics underlying bladder injury, while highlighted here, has not been sufficiently documented in the literature. Almost no data exist for children, adolescents, or even adults explaining the exact pathophysiology of isolated bladder injury. Therefore, hematuria after a lightning strike should prompt evaluation of the bladder, in addition to renal injury. Partial injuries or contusions can be managed with placement of a draining catheter, while intraperitoneal or extraperitoneal ruptures, though less likely, can be managed according to standard urological protocols in children and based on clinical stability. Early recognition and appropriate management are essential for favorable outcomes.

## Conclusions

This case underscores the importance of maintaining a high index of suspicion for internal injuries, including bladder injury, in lightning strike victims, even when external signs are minimal, especially when the arborizing pattern of the lightning injury shows atypical exit points. Lightning strikes can produce a wide spectrum of injuries, but isolated bladder rupture, particularly in children and adolescents, remains exceptionally rare and, in fact, undocumented in the available literature. This case highlights the need for heightened clinical vigilance when evaluating lightning victims, particularly those presenting with lower abdominal pain, hematuria, and atypical patterns of lightning exit wounds. The mechanism of injury is likely related to rapid intravesical pressure changes and muscle contraction when the bladder is full at the moment of exposure, though further case reports, series, and analyses are required to better understand the causative factors of injury in atypical organs such as the bladder.

Prompt recognition using appropriate imaging and timely management, whether conservative or surgical, is essential to prevent complications and ensure favorable outcomes. Greater awareness of this unusual presentation and continued reporting of similar cases will help broaden understanding of lightning-induced internal injuries and support the development of more comprehensive evaluation strategies for affected patients. Ensuring that cases of electrical or lightning current injuries receive multidisciplinary evaluation, diagnosis, and case-by-case management, based on an understanding of the physical properties of lightning, can improve patient outcomes.
